# Treatment of an intracranial aneurysm in the setting of fenestration of cranial division of the internal carotid artery: Technical considerations and a literature review

**DOI:** 10.1177/15910199241262845

**Published:** 2024-06-17

**Authors:** Shigeta Miyake, Andrew Falzon, Tze Phei Kee, Hugo Andrade, Timo Krings

**Affiliations:** 1 Department of Neurosurgery, 13155 Yokohama City University School of Medicine, Yokohama, Kanagawa, Japan; 2 Division of Neuroradiology, Joint Department of Medical Imaging, University Health Network and Toronto Western Hospital, Toronto, ON, Canada; 3 Department of Medical Imaging, University of Toronto, Toronto, ON, Canada; 4 Department of Neuroradiology, 54738 National Neuroscience Institute, Singapore; 5 Division of Neurosurgery, 26625 Toronto Western Hospital, Toronto, Canada; 6 Sprott Department of Surgery, University of Toronto, Toronto, Canada

**Keywords:** Supraclinoid internal carotid artery fenestration, cranial division, endovascular treatment, aneurysm

## Abstract

Although rare, cerebral arterial fenestration may present challenges in diagnosis and treatment. Here we present a case of a supraclinoid internal carotid artery (ICA) fenestration adjacent to an ICA aneurysm, successfully treated with balloon-assisted coil embolization. A female in her 50's presented with an acute subarachnoid hemorrhage from a ruptured left ICA-ophthalmic artery (OA) aneurysm. Digital subtraction angiography revealed a focal ICA fenestration distal to the posterior communicating artery (Pcom). The patient underwent successful coil embolization of the aneurysm using the balloon-assisted technique. No immediate hemorrhagic, thromboembolic, or neurological complications were observed. The patient was discharged in good condition after 2 weeks of hospitalization. A comprehensive literature review of 33 cases was subsequently performed to understand the characteristics of this condition. Cases involving the cranial division of the ICA forming the fenestration exhibited caliber differences significantly more frequently (p = 0.02). Embryological insights revealed distinctions between the cranial divisions of the ICA, influencing fenestration morphology and associated aneurysm formation. Endovascular treatment poses the risk of vascular injury, necessitating the identification of this variation and procedural planning.

## Introduction

**Figure 1.**
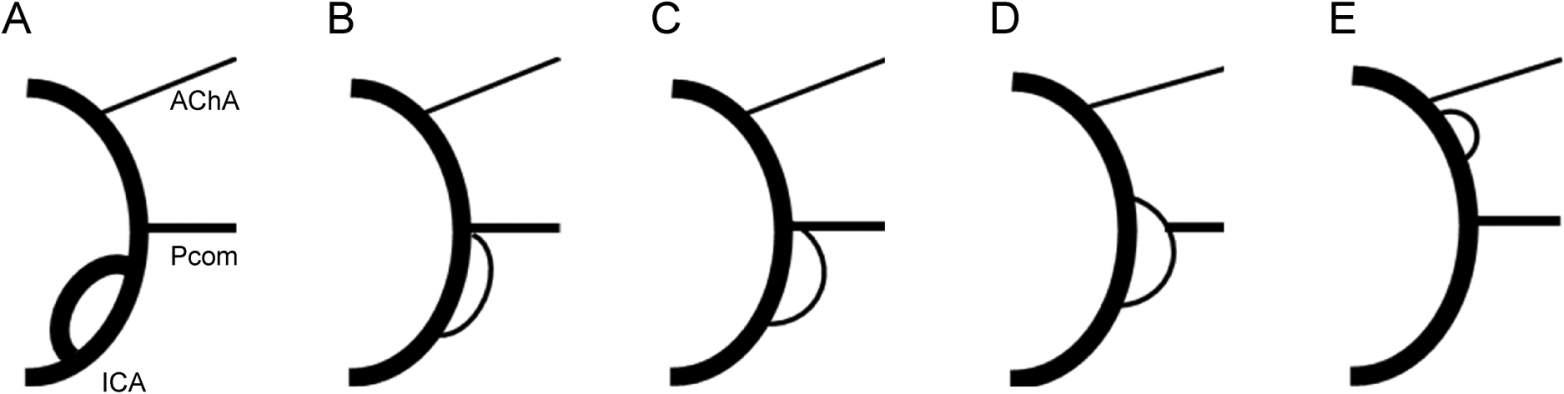
Illustrations depicting the location of the supraclinoid fenestration. A: The internal carotid artery (ICA) appears to be duplicated (equal caliber); B: Smaller limb fenestration fused to the ICA at the origin of the posterior communicating artery (Pcom); C: Smaller limb of fenestration fused to the Pcom itself; D: Smaller limb of fenestration appearing as a duplicated Pcom; E: Fenestration between the Pcom and anterior choroidal artery (AChA). Haryu et al. categorized supraclinoid ICA fenestration into three groups^2^: Type A (A), Type B (B), and Type C (C and D). Cases were assessed based on available images and descriptions in the literature. In this study, we divided the patients into two groups: one comprising the cranial division (cranial division group) (D and E), and the other located in the ICA segment proximal to the Pcom (proximal group) (A-C).

Cerebral arterial fenestration is rare, with an incidence of only 2.1% in the general population, and is notably more common in the posterior circulation.^1^ Approximately 90% of cases occur in the basilar artery (BA), vertebral artery, or anterior cerebral artery (ACA), while fenestration of the internal carotid artery (ICA) occurs in only 1.3% of cerebral arterial fenestration cases. Haryu et al. conducted a review of 16 cases of supraclinoid ICA fenestration, categorizing them into three groups^2^: Type A (where the ICA appears to be a duplicate (equal caliber)), type B (where the smaller limb of the fenestration fused to the ICA at the origin of the posterior communicating artery (Pcom)), and Type C (the smaller limb of the fenestration is fused to the Pcom itself or appears as a duplicated Pcom) (Figure 1A–D). It is noteworthy that while some Type C cases exhibit fenestrations extending beyond the Pcom (Figure 1D), ICA fenestrations between the Pcom and anterior choroidal artery (AChA) are not included in this classification (Figure 1E), with only two such cases being reported in the past.^3,4^

Generally, the etiology of cerebral arterial “fenestration” can be attributed to a lack of fusion of embryologically paired arteries (segmentally unfused arteries), two embryologically different vessels that fuse during development (duplication), or a partial residual of the embryological rete of perforators (extracerebral anastomoses between perforators).^2,5^ However, the cause of the supraclinoid ICA fenestration remains unclear. Herein, we present a case of a supraclinoid ICA fenestration located between the Pcom and AChA, accompanied by an ICA aneurysm, which was successfully treated with balloon-assisted coil embolization. This case report aims to elucidate the embryology of ICA fenestration based on a literature review and discuss the technical considerations when considering the endovascular treatment of aneurysms associated with this condition.

## Case presentation

**Figure 2.**
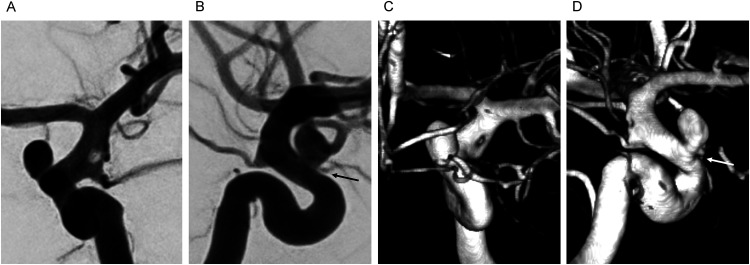
Digital subtraction angiography (DSA) with 3D reconstruction, antero-posterior (A, C), and lateral (B, D) views, demonstrating focal fenestration of the supraclinoid internal carotid artery (ICA), distal to the posterior communicating artery (Pcom), and proximal to the anterior choroidal artery (AChA) origin. The disparity in the caliber of the fenestrated vascular limbs is notable, with the posterolateral limb being smaller in caliber. The ruptured ICA-ophthalmic artery (OA) aneurysm is located distant to the fenestrated ICA segment. The OA (arrow) is incorporated into the neck of the aneurysm.

**Figure 3.**
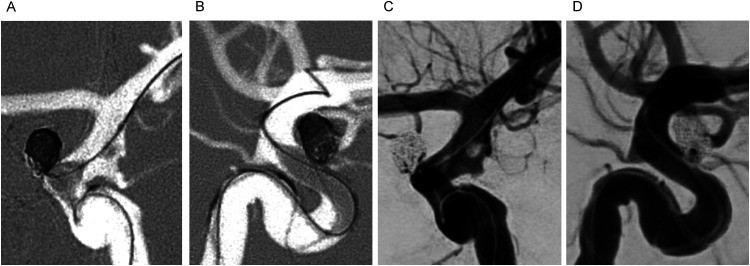
Digital subtraction angiography (DSA), antero-posterior (A, C) and lateral (B, D) views of the balloon-assisted coil embolization of the ruptured ICA-ophthalmic artery (OA) aneurysm. The balloon is positioned and inflated over the aneurysm neck, carefully sparing the fenestrated segment, with a micro guidewire through the larger fenestrated limb. Post-embolization angiogram (C, D) demonstrating near complete occlusion of the aneurysm, with flow in the OA preserved.

A female in her 50's was admitted to our hospital with an acute subarachnoid hemorrhage caused by a ruptured left ICA-ophthalmic artery (OA) aneurysm. On arrival, she presented with a Glasgow Coma Scale (GCS) score of 15, and no focal neurological deficits. Digital subtraction angiography revealed a ruptured left ICA-OA aneurysm incorporating the origin of the OA into the neck. Focal fenestration of the left supraclinoid ICA was observed between the Pcom and the AChA (Figure 2). A notable discrepancy in the caliber of the two limbs of the fenestration was observed, with the posterolateral limb being smaller in caliber. The patient was treated with balloon-assisted coil embolization of the ruptured left ICA-OA aneurysm (Figure 3), achieving satisfactory occlusion. A trace residual flow was noted at the proximal neck of the aneurysm at the site of origin of the OA, with flow preserved along the OA. Baseline postprocedural magnetic resonance imaging showed no thromboembolic complications. The patient was prescribed 81 mg of aspirin for 3 months following the procedure, and was discharged in good condition after 2 weeks of hospitalization, with no neurological deficits.

## Literature review

Table 1 summarizes the characteristics of 33 reported cases of supraclinoid ICA fenestration, including the current case and 32 additional cases identified by a literature review conducted via PubMed, using search terms including “internal carotid artery,” “posterior communicating artery,” and “fenestration.” One case was excluded because it was classified as having a duplicate origin of the Pcom. Cases were assessed based on available images and descriptions in the literature. Furthermore, considering the embryological distinction between the ICA beyond the Pcom, which originates from the cranial division of the ICA, and that proximal to the Pcom of the ICA^33^, the cases were divided into two groups: one comprising the cranial division (cranial division group) (Figure 1D and E) and the other located in the ICA segment proximal to the Pcom (proximal group) (Figure 1A–C).

**Table 1.** Comparison of cranial division and proximal lesions in supraclinoid internal carotid artery fenestration: summary of the present case and 32 previously reported cases.^2–4,6–32^

		Total	Fenestration located proximal to Pcom of ICA	Fenestration including cranial division of ICA	p value
Case		33	24 (72.7%)	9 (27.3%)	
Age (mean ± SE)		48.1 ± 2.5	47.5 ± 3.0	49.4 ± 4.6	0.73
Sex (Female)		24 (77.4%)	18 (81.8%)	6 (66.7%)	0.38
Lesion side (right)		16 (48.5%)	13 (54.17%)	3 (33.3%)	0.44
Difference in vessel caliber		21 (63.6%)	13 (54.2%)	9 (100%)	0.02
Intracranial aneurysm (cases)		28 (84.8%)	22 (91.7%)	6 (66.7%)	0.11
Subarachnoid hemorrhage		12 (38.7%)	8 (36.4%)	4 (44.4%)	0.7
Location of aneurysm	Proximal end of the fenestration	19 (67.9%)	17 (77.3%)	2 (33.3%)	0.06
	Limb of fenestration	7 (25%)	4 (18.2%)	3 (50.0%)	0.14
	Separated lesion	8 (27.6%)	5 (22.7%)	3 (42.9%)	0.36

Abbreviation: ICA: internal carotid artery, Pcom: posterior communicating artery

Age is expressed as mean ± standard error and was evaluated using a t-test. Other factors are expressed as percentages and were evaluated using Fisher's exact test. All statistical analyses were performed using JMP Pro 15 (JMP Statistical Discovery LLC, Cary, United States), with statistical significance set at p < 0.05.

The mean age of patients with supraclinoid ICA fenestration was 48.1 years, with a female predominance (77.4%), while no significant difference was observed between the sides. Additionally, 84.8% of the cases were associated with intracranial aneurysms, and 38.7% presented with aneurysmal subarachnoid hemorrhage. The cranial division group was noted in nine cases (27.3%), with no discernible differences in age, sex, or side between the groups. However, caliber differences between the two limbs forming the fenestration were significantly more common in the cranial division group (*p *= 0.02). There were no notable differences in the occurrence of intracranial aneurysms or subarachnoid hemorrhages between the groups (p = 0.11, 0.7, respectively), although a trend toward aneurysms forming at the proximal end of the fenestration in the proximal group was observed (*p *= 0.06).

## Discussions

Herein, we present the case of a woman with a ruptured left ICA-OA aneurysm proximal to the focal fenestrated ICA segment successfully treated with balloon-assisted coil embolization. Further, our literature review constitutes the largest compilation of previous reports using statistical analyses. Our review demonstrated notable caliber differences between the two fenestrated limbs if the cranial ICA division was involved. Further, our findings suggest that aneurysms related to fenestrations located proximal to the Pcom tend to develop at the proximal end of the fenestration.

### Embryological hypothesis

Embryologically, the ICA is divided into cranial and caudal segments at the 4–5 mm stage^2,33^, with the cranial division developing into the anterior circulation and the caudal division into the Pcom. Therefore, the ICA beyond the Pcom is embryologically derived from the cranial divisions of the ICA, and is distinct from the segment proximal to the Pcom. In this study, fenestrations involving the cranial division were observed to have caliber differences between the two limbs forming the fenestration; this discrepancy/asymmetry was also observed in the present case. Because cerebral arterial fenestration is considered a congenital condition^2^, the differences in the morphology of the limbs forming the fenestration can be hypothetically explained embryologically.

Generally, cerebral arterial fenestration is considered to be caused by the failure of the fusion of two equivalent vessels, such as paired ventral longitudinal neural arteries that eventually form the BA.^5,34^ In such cases, the two arteries forming the fenestration have equal caliber.^35^ Extracerebral anastomoses between perforators, in which the embryological rete of the perforators is partially residual, have also been proposed.^5^ Although rare, fenestration of the middle cerebral artery (MCA) is thought to be caused by a failure of confluency in the extinction of the plexiform arteries.^36^ In such cases, there is a difference in caliber between the two arteries that form the fenestration. Based on the two mechanisms of fenestration formation, the caliber differences in fenestrations, including cranial divisions, may be related to the presence of a plexiform stage in the formation of the AChA and MCA during ICA cranial division development.^2^ Just as the temporopolar artery bifurcations are thought to be related to the formation of smaller fenestrations in the MCA^36^, it is also possible that the AChA and perforating arteries are related to fenestration formation. The presence of a smaller posterolateral fenestration at this site supports this hypothesis.

On the other hand, if the fenestration is located proximal to the Pcom, the two limbs forming the fenestration are comparable, suggesting that arteries of comparable sizes may fuse during fetal development. The segment between the OA and the Pcom is thought to be derived from the third aortic arch during embryonic life^33^, and there are no major branching vessels in this segment. Further, the cranial and caudal divisions are thought to be of equal caliber. This suggests that fenestration in the ICA segment proximal to the Pcom occurs during development when the Pcom is formed by caudal division. The caudal division that branches off from the proximal part of the ICA fuses to a more distal portion during development, which may have caused fusion failure.

In this case, confluence failure in the plexiform extinction of the cranial division of the ICA may have contributed to the ICA fenestration. Anatomically, the oculomotor nerve is located inferolateral to the ICA, and an aneurysm in this region can cause compression of the oculomotor nerve and, in rare cases, fenestration of the oculomotor nerve.^37^ Since the oculomotor nerve runs along the dura mater between the posterior clinoid process and the oculomotor triangle,^38^ it is not thought to penetrate the ICA.

### Intracranial aneurysm formation

Cerebral arterial fenestrations are often associated with aneurysm formation.^6^ There are two main theories to explain the formation of an aneurysm in the presence of arterial fenestration: the fragility of the vascular structure in the fenestrated vessels and the hemodynamic stress at the site of fenestration.^2^ Although some studies have shown that the fragility of the vessel wall can be attributed to the loss of the muscular layer^2^, this has yet to be proven histologically in ICA.

Given that the ICA is a high-flow artery, narrowing caused by fenestration and turbulence may lead to hemodynamic stress. The high reporting rate of ICA fenestration compared to its prevalence suggests that ICA fenestration may be more prone to aneurysm formation than other sites^1^, potentially due to the high flow characteristics of the ICA. Computational fluid dynamics with the fenestration model have shown that stress on the vessel wall is localized at the proximal end of the fenestration and the distal lateral part of the limb^35^, which could explain the multifocal formation of aneurysms.

In this study, approximately 40% of supraclinoid ICA fenestrations with associated aneurysms presented with subarachnoid hemorrhage. However, as this literature review was heavily based on case reports, it is likely that the incidence of ICA fenestration and associated aneurysms was underestimated. This study also highlighted a higher tendency for aneurysms to form at the proximal end of the fenestration proximal to the Pcom group, which is presumably related to hemodynamic stress. Further research is required to study the differences in hemodynamic stress based on the location of fenestration and morphological characteristics, such as vessel caliber and incorporated arterial branches.

### Endovascular treatment

It is important to recognize ICA fenestration on preprocedural cross-sectional and initial diagnostic angiographic images, as this focal filling defect may mimic a focal thrombus or air bubble.^39^ To avoid complications, we suggest that in cases where balloon remodeling is deemed necessary, the balloon microcatheter—micro guidewire assembly should be navigated through the larger limb of the fenestration, with precise positioning of the balloon and gentle inflation over the aneurysm neck, sparing the fenestrated ICA segment to avoid vessel injury. In cases in which endovascular treatment is deemed too risk, for example because of the presence of fenestrated or deformed vessels, open surgical treatment should be considered. Vigilant post-treatment follow-up may be required because of the potentially higher risk of aneurysm recurrence or de novo aneurysm formation.

## Conclusions

Supraclinoid ICA fenestration, particularly in the cranial division, may pose diagnostic and therapeutic challenges. Embryological origins and hemodynamic stress may contribute to fenestration morphology and associated aneurysm formation. Although effective, endovascular treatment requires meticulous planning to mitigate the risk of vascular injury. Our findings further underscore the importance of a precise diagnosis and careful procedural execution in managing this rare but clinically significant condition.

## Abbreviations

ACA: anterior cerebral artery
AChA: anterior choroidal artery
BA: basilar artery
ICA: internal carotid artery
MCA: middle cerebral artery
OA: ophthalmic artery
Pcom: posterior communicating artery

## References

[1] Cooke DL Stout CE Kim WT , et al. Cerebral arterial fenestrations. Interv Neuroradiol 2014; 20: 261–274. 24976087 10.15274/INR-2014-10027 PMC4178766

[2] Haryu S Sato K Matsumoto Y , et al. Supraclinoid internal carotid artery fenestration with associated aneurysm: case report and literature review. NMC Case Rep J 2020; 7: 183–187. 33062566 10.2176/nmccrj.cr.2019-0301 PMC7538463

[3] Hattori T Kobayashi H . Fenestration of the supraclinoid internal carotid artery associated with carotid bifurcation aneurysm. Surg Neurol 1992; 37: 284–288. 1595041 10.1016/0090-3019(92)90154-f

[4] Rennert J Ullrich WO Schuierer G . A rare case of supraclinoid internal carotid artery (ICA) fenestration in combination with duplication of the middle cerebral artery (MCA) originating from the ICA fenestration and an associated aneurysm. Clin Neuroradiol 2013; 23: 133–136. 22231576 10.1007/s00062-011-0120-3

[5] Krings T Baccin CE Alvarez H , et al. Segmental unfused basilar artery with kissing aneurysms: report of three cases and literature review. Acta Neurochir (Wien) 2007; 149: 567–574; discussion 574. 17514352 10.1007/s00701-007-1118-0

[6] van Rooij SB van Rooij WJ Sluzewski M , et al. Fenestrations of intracranial arteries detected with 3D rotational angiography. AJNR Am J Neuroradiol 2009; 30: 1347–1350. 19439481 10.3174/ajnr.A1563 PMC7051541

[7] Yock DHJ . Fenestration of the supraclinoid internal carotid artery with rupture of associated aneurysm. AJNR Am J Neuroradiol 1984; 5: 634–636. 6435433 PMC8335121

[8] Findlay JM Chui M Muller PJ . Fenestration of the supraclinoid internal carotid artery. Can J Neurol Sci 1987; 14: 159–161. 3607619 10.1017/s0317167100026317

[9] Takano S Saitoh M Miyasaka Y , et al. Fenestration of the intracranial internal carotid artery—case report. Neurol Med Chir (Tokyo) 1991; 31: 740–742. 1723165 10.2176/nmc.31.740

[10] Banach MJ Flamm ES . Supraclinoid internal carotid artery fenestration with an associated aneurysm. Case report. J Neurosurg 1993; 79: 438–441. 8360743 10.3171/jns.1993.79.3.0438

[11] Katsuta T Matsubara T Fujii K . Fenestration of the supraclinoid internal carotid artery. Neuroradiology 1993; 35: 461. 8377923 10.1007/BF00602832

[12] Tripathi M Goel V Padma MV , et al. Fenestration of the posterior communicating artery. Neurol India 2003; 51: 75–76. 12865525

[13] Ng PP Steinfort B Stoodley MA . Internal carotid artery fenestration with dual aneurysms. Case illustration. J Neurosurg 2006; 104: 979. 16776346 10.3171/jns.2006.104.6.979

[14] Bharatha A Fox AJ Aviv RI , et al. CT Angiographic depiction of a supraclinoid ICA fenestration mimicking aneurysm, confirmed with catheter angiography. Surg Radiol Anat 2007; 29: 317–321. 17429569 10.1007/s00276-007-0205-5

[15] Onoda K Ono S Tokunaga K , et al. Fenestration of the supraclinoid internal carotid artery with associated aneurysm. Neurol Med Chir (Tokyo) 2008; 48: 118–120. 18362458 10.2176/nmc.48.118

[16] Baba S Fukuda Y Mizota S , et al. Fusiform aneurysm associated with fenestration of the posterior communicating artery. Neurol Med Chir (Tokyo) 2010; 50: 568–570. 20671382 10.2176/nmc.50.568

[17] Plumb AA Herwadkar A Pickett G . Incidental finding of fenestration of the supraclinoid internal carotid artery with appearances on magnetic resonance angiography. Surg Radiol Anat 2010; 32: 165–169. 19756351 10.1007/s00276-009-0555-2

[18] Dey M Awad IA . Fenestration of supraclinoid internal carotid artery and associated aneurysm: embryogenesis, recognition, and management. World Neurosurg 2011; 76: 592.e1–592.e5. 10.1016/j.wneu.2011.04.019 22251509

[19] Ichikawa T Miyachi S Izumi T , et al. Fenestration of a supraclinoid internal carotid artery associated with dual aneurysms: case report. Neurosurgery 2011; 69: E1005–E1008; discussion E1009. 10.1227/NEU.0b013e318223b613 21572363

[20] Nakiri GS Bravo E Al-Khawaldeh M , et al. Endovascular treatment of aneurysm arising from fenestration of the supraclinoid internal carotid artery—two case reports. J Neuroradiol 2012; 39: 195–199. 22189288 10.1016/j.neurad.2011.10.001

[21] Park SH Lee CY . Supraclinoid internal carotid artery fenestration harboring an unruptured aneurysm and another remote ruptured aneurysm: case report and review of the literature. J Cerebrovasc Endovasc Neurosurg 2012; 14: 295–299. 23346545 10.7461/jcen.2012.14.4.295 PMC3543915

[22] Orru E Wyse E Pearl MS . Fenestration of the supraclinoid internal carotid artery in a patient with a concomitant intracranial arteriovenous malformation. BMJ Case Rep 2015; 2015. 10.1136/bcr-2015-209733 PMC4434274 25948856

[23] Weiner GM Grandhi R Zwagerman NT , et al. Aneurysmal subarachnoid hemorrhage with concomitant posterior communicating artery fenestration. Int J Neurosci 2015; 125: 154–158. 24761761 10.3109/00207454.2014.918119

[24] Uchino A Tanaka M . Fenestration of the supraclinoid internal carotid artery arising from the paraclinoid aneurysmal dilatation and fusing with the origin of the posterior communicating artery: a case report. Surg Radiol Anat 2017; 39: 581–584. 27695969 10.1007/s00276-016-1753-3

[25] Jha N Crockett MT Singh TP . Unusual right internal carotid artery supraclinoid segment fenestration associated with multiple aneurysms treated with flow diversion and coiling. BMJ Case Rep 2018; 2018. 10.1136/bcr-2018-227020 PMC6144410 30206070

[26] Lee GY Shin GW Jung HS , et al. Fenestration of the supraclinoid internal carotid artery connecting the neck of the paraclinoid aneurysm and the origin of the posterior communicating artery: a case report. Interv Neuroradiol 2018; 24: 274–276. 29383975 10.1177/1591019917753825 PMC5967182

[27] Sgreccia A Coskun O Di Maria F , et al. Fenestration of the supraclinoid segment of the ICA and associated aneurysms: a case report with literature review. Acta Neurochir (Wien) 2018; 160: 1143–1147. 29675721 10.1007/s00701-018-3551-7

[28] Kasper J Nestler U Meixensberger J , et al. Treatment of supraophthalmic internal carotid artery fenestration with an associated aneurysm via flow diversion: a case report. Int Med Case Rep J 2021; 14: 487–491. 34321932 10.2147/IMCRJ.S317709 PMC8309652

[29] Schmidt RF Sweid A Chalouhi N , et al. Endovascular management of complex fenestration-associated aneurysms: a single-institution retrospective study and review of existing techniques. World Neurosurg 2021; 146: e607–e617. 10.1016/j.wneu.2020.10.131 33130285

[30] Filep RC Constantin C Arbǎnaṣi EM , et al. Endovascular treatment of an aneurysm associated with fenestration of the supraclinoid internal carotid artery: case report and review of the literature. Front Neurol 2022; 13: 966642. 36438971 10.3389/fneur.2022.966642 PMC9682165

[31] Uchino A . Supraclinoid internal carotid artery fenestration from which the posterior communicating artery arising with infundibular dilatation at its origin diagnosed by magnetic resonance angiography. Radiol Case Rep 2022; 17: 2579–2582. 35634016 10.1016/j.radcr.2022.04.033 PMC9130076

[32] Zhou Z Yu J . Endovascular treatment of a supraclinoid internal carotid artery fenestration aneurysm: a case report and literature review. Heliyon 2023; 9: e17605. 10.1016/j.heliyon.2023.e17605 PMC10318508 37408880

[33] Lasjaunias P Santoyo-Vazquez A . Segmental agenesis of the internal carotid artery: angiographic aspects with embryological discussion. Anat Clin 1984; 6: 133–141. 6498000 10.1007/BF01773165

[34] Tsuei YS Matsumoto Y Ohta M , et al. Vertebrobasilar junction fenestration with dumbbell-shaped aneurysms formation: computational fluid dynamics analysis. Surg Neurol 2009; 72: S11–S19. 10.1016/j.surneu.2009.05.026 19664810

[35] Tong X Dong J Zhou G , et al. Hemodynamic effects of size and location of basilar artery fenestrations associated to pathological implications. Int J Numer Method Biomed Eng 2021; 37: e3507. 10.1002/cnm.3507 34184422

[36] Gailloud P Albayram S Fasel JH , et al. Angiographic and embryologic considerations in five cases of middle cerebral artery fenestration. AJNR Am J Neuroradiol 2002; 23: 585–587. 11950648 PMC7975097

[37] Binning MJ Couldwell WT . Fenestration of the oculomotor nerve by a duplicated posterior cerebral artery and aneurysm. Case report. J Neurosurg 2009; 111: 84–86. 19284238 10.3171/2009.2.JNS081688

[38] Martins C Yasuda A Campero A , et al. Microsurgical anatomy of the oculomotor cistern. Neurosurgery 2006; 58. doi:10.1227/01.NEU.0000204673.55834.BE. 16582644

[39] Sakai Y Yoshikawa G Sato K . Mechanical thrombectomy for ICA top occlusion with twig-like MCA: a case report. J Neuroendovasc Ther 2022; 16: 175–180. 37502284 10.5797/jnet.cr.2020-0202 PMC10370778

